# Mg Doped Li–LiB Alloy with In Situ Formed Lithiophilic LiB Skeleton for Lithium Metal Batteries

**DOI:** 10.1002/advs.201902643

**Published:** 2020-01-09

**Authors:** Chen Wu, Haifeng Huang, Weiyi Lu, Zengxi Wei, Xuyan Ni, Fu Sun, Piao Qing, Zhijian Liu, Jianmin Ma, Weifeng Wei, Libao Chen, Chenglin Yan, Liqiang Mai

**Affiliations:** ^1^ State Key Laboratory of Powder Metallurgy Central South University Changsha 410083 China; ^2^ School of Physics and Electronics Hunan University Changsha 410082 China; ^3^ College of Energy Key Laboratory of Advanced Carbon Materials and Wearable Energy Technologies of Jiangsu Province Key Laboratory of Advanced Optical Manufacturing Technologies of Jiangsu Province, and Key Laboratory of Modern Optical Technologies of Education Ministry of China Soochow University Suzhou 215006 China; ^4^ Qingdao Institute of Bioenergy and Bioprocess Technology Chinese Academy of Sciences Qingdao 266101 China; ^5^ State Key Laboratory of Advanced Technology for Materials Synthesis and Processing Wuhan University of Technology Wuhan 430070 China

**Keywords:** buffer volume change, dendrite‐free, Li–B–Mg composite, lithiophilic 3D skeleton, ultralong lifespan

## Abstract

High energy density lithium metal batteries (LMBs) are promising next‐generation energy storage devices. However, the uncontrollable dendrite growth and huge volume change limit their practical applications. Here, a new Mg doped Li–LiB alloy with in situ formed lithiophilic 3D LiB skeleton (hereinafter called Li–B–Mg composite) is presented to suppress Li dendrite and mitigate volume change. The LiB skeleton exhibits superior lithiophilic and conductive characteristics, which contributes to the reduction of the local current density and homogenization of incoming Li^+^ flux. With the introduction of Mg, the composite achieves an ultralong lithium deposition/dissolution lifespan (500 h, at 0.5 mA cm^−2^) without short circuit in the symmetrical battery. In addition, the electrochemical performance is superior in full batteries assembled with LiCoO_2_ cathode and the manufactured composite. The currently proposed 3D Li–B–Mg composite anode may significantly propel the advancement of LMB technology from laboratory research to industrial commercialization.

## Graphical abstract

A Li–B–Mg composite with in situ formed 3D LiB fiber network shows a dendrite‐free morphology and less volume change during cycling. The symmetrical battery achieves a long and stable cycle lifespan of more than 500 h at 0.5 mA cm^−2^ due to the effect of skeleton and the addition of Mg. The full battery also displays improved electrochemical performance.

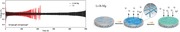

## 1 Introduction

The advent of lithium ion batteries (LIBs) has significantly mitigated the crisis of fossil fuels^1^ and they have become an integral part of our modern life by ubiquitously powering various of modern electronics such as mobile phones, laptops, electric vehicles (EVs), unmanned aircraft, etc. However, the state‐of‐the‐art LIBs dominating the market can hardly satisfy the soaring need for next‐generation energy storage devices of higher energy density.^2^ Superior electrode material and advanced battery technology are critically required. Recently, lithium metal anode has drawn tremendous attention worldwide due to its low potential (−3.04 V vs SHE) and ultrahigh specific capacity (3860 mA h g^−1^, 10 times as that of graphite)^3^ and it has been generally acknowledged that it is the most promising anode candidate for next‐generation high energy density batteries. In fact, it was used as an important anode for primary batteries in early 1970s. Nevertheless, several main issues summarized as follows still impede its further commercialization as an essential secondary battery component. The first one is the infinite volume change due to its “hostless” feature, which would inevitably lead to pulverization of the electrode and the failure of batteries. The second one is the formation of Li dendrite, which is the main cause for internal short circuit, decreased cycle life expectancy and low Coulombic efficiency.^4^, ^5^ Besides, the irreversible reaction between Li and nonaqueous electrolyte also consumes large amount of Li and electrolyte.^6^, ^7^ Overall, the development of Li anode faces enormous challenges.

Breakthroughs in improving the electrochemical performance of Li metal anode may bring a renaissance of the lithium metal battery (LMB) technology. To this end, researchers have explored many strategies to improve the electrochemical performance of Li metal anode such as interfacial modifications,^8^ engineered anodes,^9^, ^10^, ^11^ artificial solid electrolyte interface (SEI) films,^7^, ^12^ protective coating layers,^13^ and chemical pretreatments.^14^ In addition, a number of theoretical models were also built to describe the nucleation and growth mechanisms of Li dendrite.^15^ Chazalviel's model describes the inversely proportional relationship between the time of dendrite growth and the current density (τ ≈ J^−2^). According to this theory, constructing 3D skeleton/Li composite to substitute Li is an effective strategy to improve cycle lifespan because the 3D skeleton can not only reduce local current density and guide homogeneous Li deposition, but also minimize the volume change, which cannot be accomplished by other modification ways.^16^ Tremendous efforts on designing and constructing various Cu‐ and C‐based 3D skeleton current collectors were proposed.^11^, ^17^ However, these two kinds of skeletons are not lithiophilic. Moreover, the Li‐free characteristic of these skeletons makes them complicated for their application in Li–O_2_ and Li–S batteries. A more suitable and technically simple 3D skeleton/Li composite anode for LMBs is highly desirable.

Herein a new smelting reaction generated 3D Mg doped Li–LiB alloy (hereinafter called Li–B–Mg composite), which is mainly composed of LiB skeleton and free Li with a few dissolved Mg, is presented as a promising alternative anode for LMBs. The unique 3D composite microstructure endows the Li–B–Mg composite with several advantages. First, the 3D LiB compound fiber skeleton which is in situ formed acts as a host for Li plating/stripping, so significant volume variation could be reduced during the Li electrochemical dissolution/deposition process. Second, the superlithiophilic conductive 3D skeleton also plays an important role in retarding Li dendrites growth owing to the reduced local current density and the homogeneous regulation of Li^+^ deposition. Third, the addition of Mg element to the 3D LiB skeleton fundamentally enhances the adsorption energy of Li according to the density functional theory (DFT) calculation. It has been reported that the Li(Mg) alloy could not only produce a lower interfacial resistance, but also maintain the structural integrity of the 3D Li–B–Mg composite architecture.^18^, ^19^ Therefore, the Li–B–Mg composite showed dendrite‐free morphology and less volume variation during battery cycling. The full battery composed of the Li–B–Mg composite anode and LiCoO_2_ cathode performed a longer cycle lifespan than that of the pure Li metal. It is also worth mentioning that large‐scale fabrication of Li–B–Mg composite with a tailored structure can be achieved, providing a favorable support for its potential industrialization.

## 2 Results and Discussion

### 2.1 Characteristics of Li–B–Mg Composite

**Figure 1:**
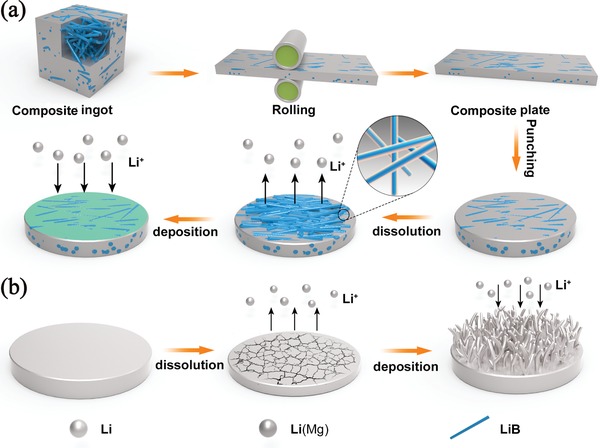
a) The schematic diagram for the fabrication course and the Li electrodissolution/electrodeposition process of Li–B–Mg composite. b) The Li electrodissolution/electrodeposition process of pure Li foil.

The schematic diagram (**Figure** **1**a) depicts the processing course of the Li–B–Mg composite as well as the Li electrodissolution/electrodeposition behavior during electrochemical cycling. Figure 1b schematically shows the electrodissolution/electrodeposition behavior of pure Li during electrochemical cycling. From Figure 1a it can be noted that the synthesized composite ingot is mainly composed of randomly oriented LiB fiber skeleton that is in situ formed and free Li (Mg solubilizes in Li phase, marked as Li(Mg)). The composite ingot has been densified after rolling and the density of the composite plate is measured to be 0.844 g cm^−3^. During the rolling process, some LiB fibers may align roughly in parallel along with the rolling direction. When Li is electrochemically dissolved, the skeleton maintains the integrity of the composite anode while in the reverse process it regulates the electrochemical deposition of Li and inhibits dendrite formation. In addition, the introduction of Mg forms Li(Mg) solid solution filled in LiB skeleton. Besides the enhanced affinity to Li and regulation of Li^+^ flux, it could transform into a Li‐deficient phase during Li stripping process and connect the fibers, contributing to the stability of the electrode. In comparison, Figure 1b shows that pure Li foil would experience large volume change and inevitable growth of dendritic Li during electrochemical cycling could occur.

**Figure 2:**
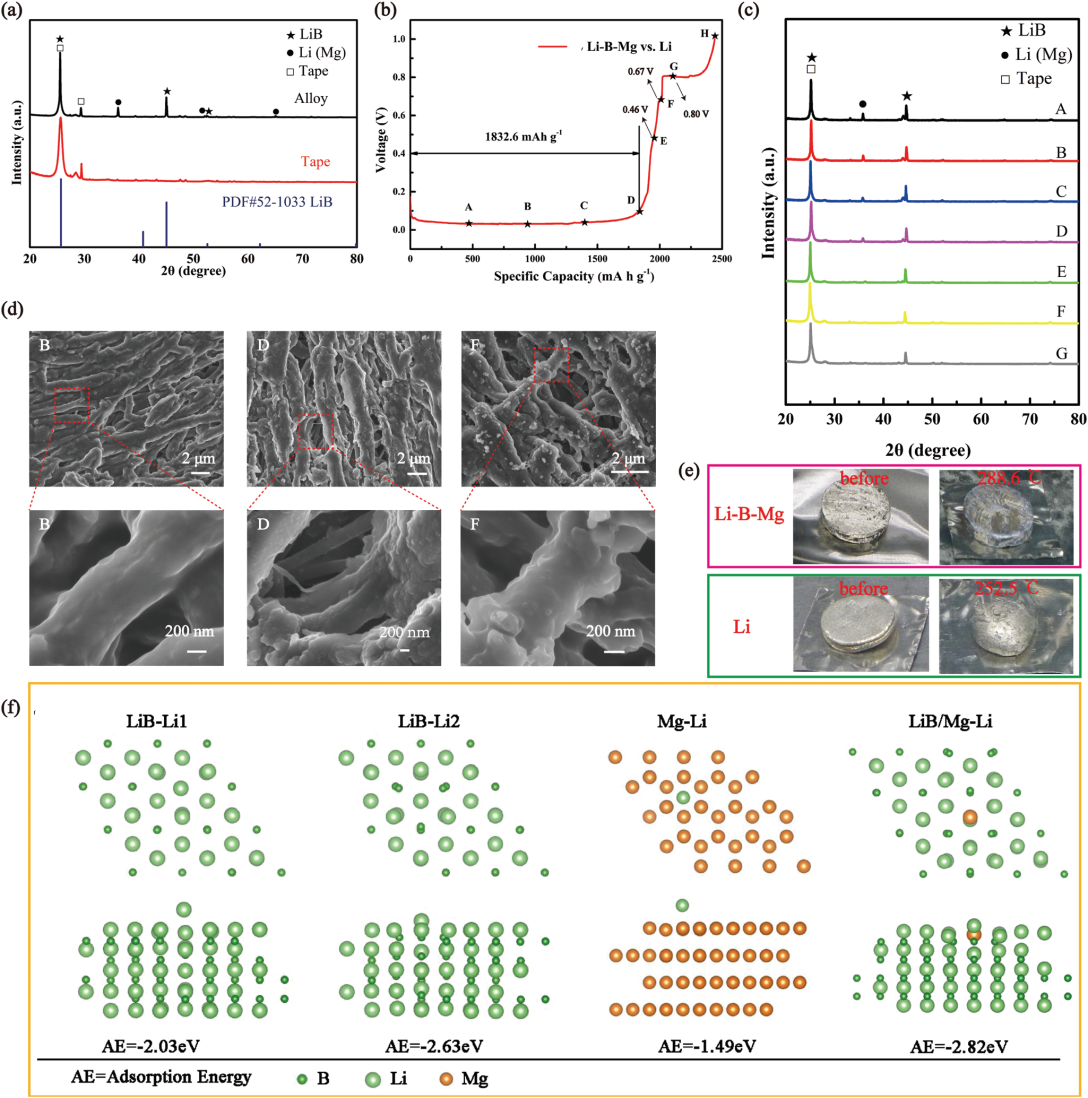
a) XRD results of the obtained 3D Li–B–Mg composite. b) Voltage profile of the electrochemical Li stripping from the composite anode to 1 V versus Li^+^/Li. c) The corresponding XRD patterns and d) SEM images of the composite at specific stages marked in (b). e) Study of the structural stability of the 3D Li–B–Mg composite at high temperature. f) Calculations of adsorption energies of a Li atom on the surface of LiB, Mg, and LiB/Mg.

The properties of the obtained Li–B–Mg composite have been further characterized. Figure S1 (Supporting Information) shows that the Li–B–Mg composite plate has a yellowish metallic sheen color while the pure Li has a silver color. **Figure** **2**a shows the X‐ray powder diffraction (XRD) results of the Li–B–Mg composite, from which three main peaks located at 25.53°, 45.02°, and 52.48° can be assigned to LiB phase (PDF#52‐1033, note that the disappearance of the peak at 40.77° of LiB phase is caused by the rolling process, which is in agreement with previous report.^20^). Peaks at 25.53° and 29.36° can be assigned to the tape used for the sample preparation. The remaining three peaks (at 36.16°, 52.08°, and 65.05°) belong to Li(Mg) solid solution (approximately Li_0.98_Mg_0.02_, for details of the calculation see the Supporting Information). The composite is thus confirmed to be composed of two phases, i.e., the LiB compound and Li(Mg) solid solution. In addition, the mass percent of the LiB in the composite is calculated to be 42.693 wt% (see details in the Supporting Information). Overall, the structure of the obtained Li(Mg) solid solution filled in LiB fibers skeleton is similar to that of pure Li (PDF#15‐0401) which possesses a body‐centered cubic phase. The similarities of the structure between the Li(Mg) solid solution and Li could in theory avoid phase transformation during Li electrodissolution/electrodeposition, which facilitates the structural stability of the obtained Li–B–Mg composite.^21^, ^22^, ^23^

The 3D LiB skeleton structure was observed by scanning electron microscopy (SEM) after soaking the Li–B–Mg composite in naphthalene (10 wt. %)‐containing tetrahydrofuran (THF) solution to dissolve the Li component.^24^ Figure S2a (Supporting Information) shows that the remaining 3D plate structure possesses pores of several micros in size and that the remaining LiB fibers align in parallel along the rolling direction, in accordance with the previous reports.^25^ In addition, it can be seen from Figure S2b (Supporting Information), the cross‐section of the plate, that LiB fibers of different lengths were interlaced with one another mainly in a direction parallel to the surface. The pores between LiB skeletons are thus the ideal accommodations for free Li encapsulation.

To examine the practical capacity of the prepared Li–B–Mg composite anode, it has been charged at 0.2 mA cm^−2^ in a Li‐half battery and the voltage profile result is shown in Figure 2b. From Figure 2b, a long stage below 0.1 V, which can be assigned to the stripping of free Li, can be observed. This capacity is calculated to be 1832 mA h g^−1^ based on the mass of the whole composite anode. In addition, three small platforms located at 0.46, 0.67, and 0.80 V, which can be attributed to the Li electrodissolution from the LiB compound, can be observed.^26^ To further examine the compositional and morphological change of the 3D Li–B–Mg composite anode during Li electrodissolution, ex situ XRD and SEM measurements have been conducted during the charge process at specific stages (marked from A–H in Figure 2b) and the corresponding results are shown in Figure 2c,d. From Figure 2c, it can be observed that one of the XRD peaks of the Li(Mg) solid solution, which is located at 36°, gradually decreases during Li electrodissolution process (Figure S3a, Supporting Information). This implies that the original Li(Mg) solid solution which has filled in the pores of the fibrous LiB skeleton has gradually transformed into a Li‐deficient phase during the charge process. Meanwhile, Figure 2c shows that the XRD peaks belonging to the LiB compound, which is located at 45.06°, rise first and then fall during Li electrodissolution. This indicates that the Li in the LiB compound could be dissolved to some extent during the charge process. It has to be noted that although the Li in the LiB compound can contribute extra capacity during the charge process, yet it will jeopardize the structural integrity of the LiB compound structure, resulting in an unstable 3D Li–B–Mg composite anode. The deterioration of the LiB compound can be observed via the appearance of the dark LiB color as shown in Figure S3b (Supporting Information, see also below) especially after the voltage reaches greater than 0.8 V.^10^

The Li–B–Mg composite anode which has been charged to several specific stages (B, D, and F in Figure 2b) is chosen to study its morphology changes and the corresponding results are shown in Figure 2d. One can clearly observe the appearance of large pores among the LiB fibers during the Li electrochemical dissolution under the voltage of 0.1 V (B panel in Figure 2d). With almost all available Li in Li(Mg) solid solution dissolved (D panel in Figure 2d), the pores among the LiB fibers became larger. The surface morphology and the cross‐sectional SEM images of the Li–B–Mg composite anode after Li in the LiB compound has been partially dissolved are shown in Figure 2d (F panel) and Figure S3c (Supporting Information). Moreover, one can clearly observe the fragmentation of the Li–B–Mg composite anode after the voltage has increased to 1 V, as shown in Figure S3d (Supporting Information; H point in Figure 2d). The unambiguous observation provides supporting evidence that the structure of the obtained 3D Li–B–Mg composite could be significantly damaged after the stripping of Li in LiB skeleton.^23^ On the basis of the measurements, the capacity below 0.1 V (D point) is defined as the available free Li capacity (≈1832 mA h g^−1^) and the cut‐off voltage of the following electrochemical measurement of the battery is set to be 0.4 V to avoid the destruction of LiB skeleton.

High temperature melting test and DFT calculation have been further conducted to investigate the structural stability of the obtained 3D Li–B–Mg composite. The results are respectively shown in Figure 2e,f. Figure 2e clearly shows the intact structure of the Li–B–Mg composite plate (no free Li melts out) even when it is heated to a high temperature of 288.6 °C. In comparison pure Li has melted into a ball shape under this high temperature condition. This investigation clearly demonstrates a strong structural stability under high temperature condition. To further study the chemical affinity between lithium alloy skeleton and lithium, we calculated the adsorption energies of Li on different sites and substrates by using the DFT and the results are illustrated in Figure 2f. From Figure 2f, it can be seen that the adsorption energy is −2.03 eV between a Li atom and the top Li atom of the LiB skeleton. However, when Li is adsorbed on the top of B atom in the LiB, the adsorption energy has decreased to −2.63 eV, which suggests a stronger adsorption preference in the LiB substrate (LiB–Li2). The strong interaction between LiB and Li facilitates the absorption of Li^+^ by the LiB skeleton, highlighting its lithiophilicity and availability of the host. The influence of Mg toward the affinity to Li has been also studied. In the first place the adsorption energy between Li atom and the unoccupied site and the bridge site of Mg metal are found respectively to be −1.49 and −1.48 eV. After Mg was introduced into the LiB compound, the calculation indicates an enhanced adsorption energy of Li (by about 0.19 eV relative to the adsorption energy (LiB–Li2) of −2.63 eV). The results declare an important role played by Mg atom and the enhanced chemical affinity of Li to the skeleton in the presence of Mg is thus obtained.

To further elaborate the effect of Mg, the Li–B composite was synthesized without adding Mg during the preparation process. The component contents of Li–B alloy are 74 wt.% Li and 26 wt.% B. The obtained Li–B alloy is consisted of two phases of LiB compound and free Li according to XRD patterns in Figure S4a (Supporting Information). The practical available capacity of the prepared Li–B anode (below 0.1 V) is calculated to be 1890.4 mA h g^−1^ based on the whole composite, which is a little higher than that of Li–B–Mg composite. After the free Li stripped (A stage marked in Figure S4b in the Supporting Information), the morphology of Li–B compound was shown in Figure S4c (Supporting Information). Compared with Li–B–Mg composite, the surface of LiB fibers is smoother with less adhesion after the free Li stripped (D stage) in Figure 2d. It demonstrates that after free Li stripped from Li–B–Mg, the remaining Li‐deficient Li(Mg) alloy helps connect LiB fibers to stabilize the whole skeleton. This is one of the advantage of addition of Mg.

### 2.2 Mitigation of the Volume Change

**Figure 3:**
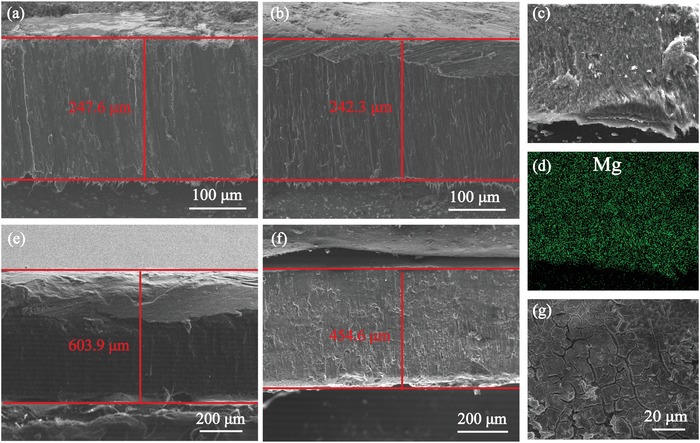
a–d) The cross‐sectional SEM images and EDX mapping of Mg of Li–B–Mg composite in different states. a) Pristine, b) after 15.3 mA h of Li was stripped, and c,d) SEM and Mg distribution after 15.3 mA h of Li was stripped. e) The pristine Li foil, f) after 24.3% Li (60 mA h of Li) was stripped, and g) the surface of Li foil after 24.3% Li was stripped.

The in situ formed lithiophilic 3D skeleton is designed to function as a “host” for Li metal to maintain the structural integrity and stability of the electrode. The ability to alleviate the significant volume change is verified through the cross‐sectional SEM, as shown in **Figure** **3**. It has to be noted that the experiment adopted about 0.2 mm thick Li–B–Mg composite plate and 0.6 mm thick Li foil. As displayed in Figure 3a, the thickness of the pristine Li–B–Mg is measured to be 247.6 µm. After 15.3 mA h of Li (25% free Li in Li–B–Mg composite) was stripped from the composite, the thickness of the composite plate decreased only by 5.3 µm (Figure 3b), less than the theoretical value of 37.11 µm (calculation details are shown in the Supporting Information). Besides, the even Mg distribution in the cross‐section of the composite after Li stripped (Figure 3c,d) corroborates that Mg remains homogeneous in the whole stripping process. It is noted that Li(Mg) alloy could act as a dual‐conductive host for Li due to the transition to a Li‐deficient material.^23^ As the concentration of Mg increases with Li stripped, the strength of the composite is enhanced, and it helps to protect the Li in LiB from being stripped. So the exist of Mg contributes to the stability of the electrode. In contrast, Figure 3e,f show that the “hostless” Li foil experienced a vast reduction in thickness of 149.3 µm after 60 mA h Li has been stripped (24.3% of the total Li corresponding to a theoretical thickness of 145.55 µm, see detailed calculations in the Supporting Information). Figure 3g shows a cracked Li surface which may result from the large volume variation during extraction of Li, as schematically illustrated in Figure 1b. The inhomogeneous Li electrodissolution/electrodeposition would lead to the observed Li electrode pulverization. In a word, the current experiment verifies that LiB skeleton can work as a scaffold to alleviate the volume variation and support the whole electrode after Li has been stripped.

### 2.3 Symmetrical Battery Performance of Li–B–Mg Composite and Li

**Figure 4:**
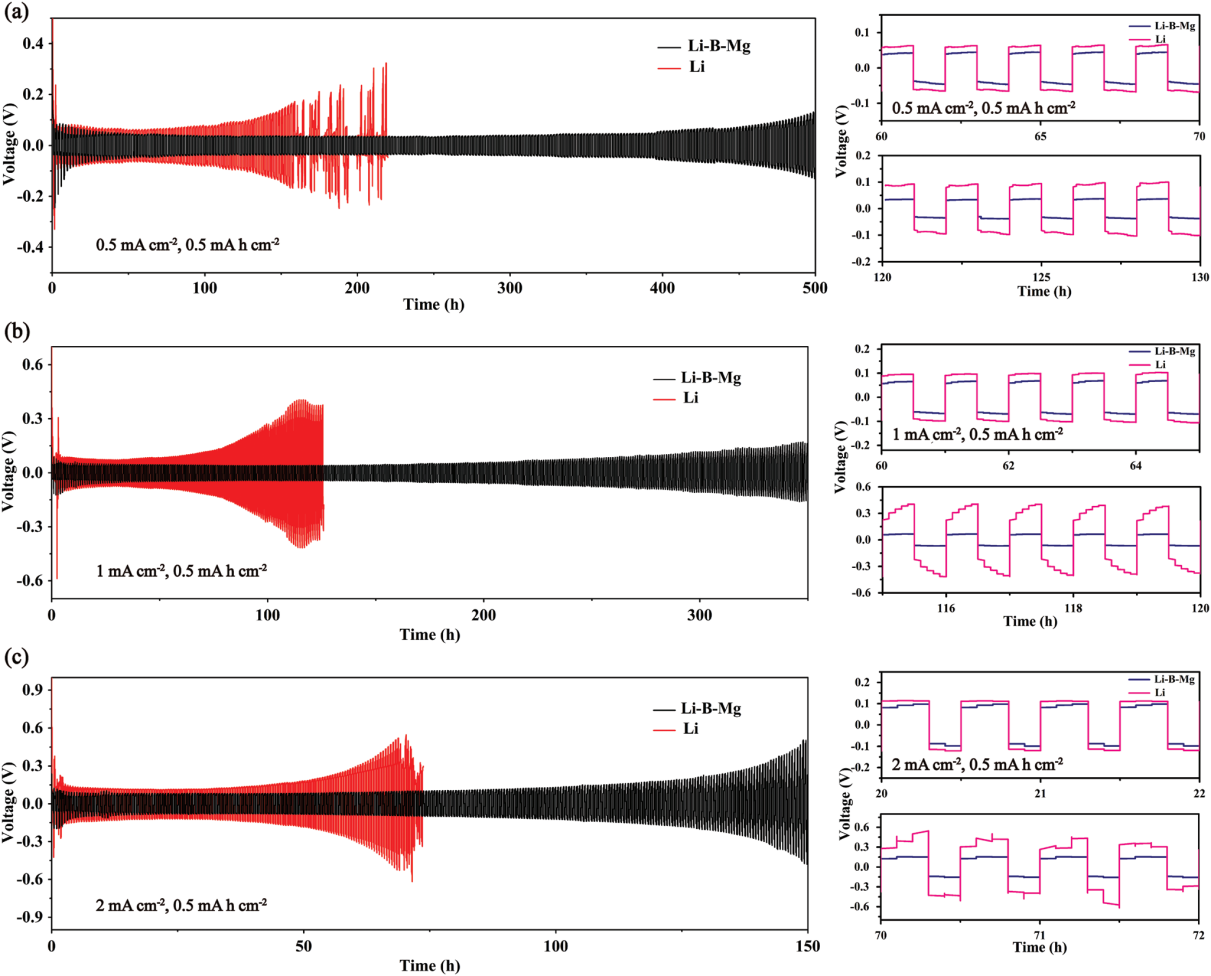
The voltage–time profiles and detailed overpotential curves of the Li–B–Mg composite assembled symmetrical batteries and pure Li assembled symmetrical batteries at a) 0.5 mA cm^−2^, b) 1 mA cm^−2^, and c) 2 mA cm^−2^ at a fixed Li stripping/plating capacity of 0.5 mA h cm^−2^.

The electrochemical performance of the symmetrical batteries assembled with either Li–B–Mg composite or pure Li is compared in **Figure** **4**. Batteries of both types were cycled at 0.5, 1, and 2 mA cm^−2^ with a fixed capacity of 0.5 mA h cm^−2^. As displayed in Figure 4a, the symmetrical battery of Li–B–Mg composite experiences an activation process at first with a large overpotential due to a few contaminants on the surface, but it quickly tends to be stable with a smaller voltage overpotential of less than 50 mV. The duration of cycle life for the Li–B–Mg symmetrical battery exceeds 500 h, whereas pure Li foil assembled battery exhibits an increasing voltage overpotential and short‐circuited after 170 hours cycling. The cycle lives of both types of batteries reduce when the current density is increased to 1 and 2 mA cm^−2^, as shown in Figure 4b,c. Nevertheless, the Li–B–Mg composite assembled symmetrical batteries show superior cycle lifespan compared with the pure Li assembled symmetrical batteries (Figure 4b,c), illustrating a stable interface and impedance formed by the Li–B–Mg composite anode.^19^ Detailed comparisons of the overpotentials are depicted in the right column of Figure 4. It has been reported that the poor performance of Li symmetrical battery results from the large volume change and formation of dendritic Li.^5^ On the contrary, the currently proposed 3D Li–B–Mg composite anode could not only reduce the local current density and mitigate the large volume change, but also regulate the uniformity of electric field (the electric field distribution is simulated hereinafter) and induce the homogeneous Li^+^ deposition. All these merits contribute tremendously to the long and stable cycling lifespan of Li–B–Mg composite assembled symmetrical batteries.

Figure S5 (Supporting Information) compares the electrochemical properties of the Li–B–Mg and Li–B composites in symmetrical batteries under the current density of 1 mA cm^−2^. It is obvious that the overpotential of Li–B–Mg composite is lower than that of Li–B anode especially in the partial enlarged figures, which is consistent with the above calculation results of enhanced adsorption energy with the addition of Mg.

The electrochemical performance of the Li–B–Mg composite in the ether‐based electrolyte was also explored since the ether‐based electrolyte is widely employed in Li–S and Li–O_2_ batteries. Figure S6 (Supporting Information) clearly shows the enhanced cycling stability of the 3D Li–B–Mg composite cycled at 1 mA cm^−2^ (over 500 h) and 2 mA cm^−2^ (250 h) compared with that of the pure Li assembled symmetrical batteries. This study further verifies the stability of the Li–B–Mg composite as a metal electrode.

### 2.4 Inhibition Mechanisms of Li Dendrite Growth During Cycling

**Figure 5:**
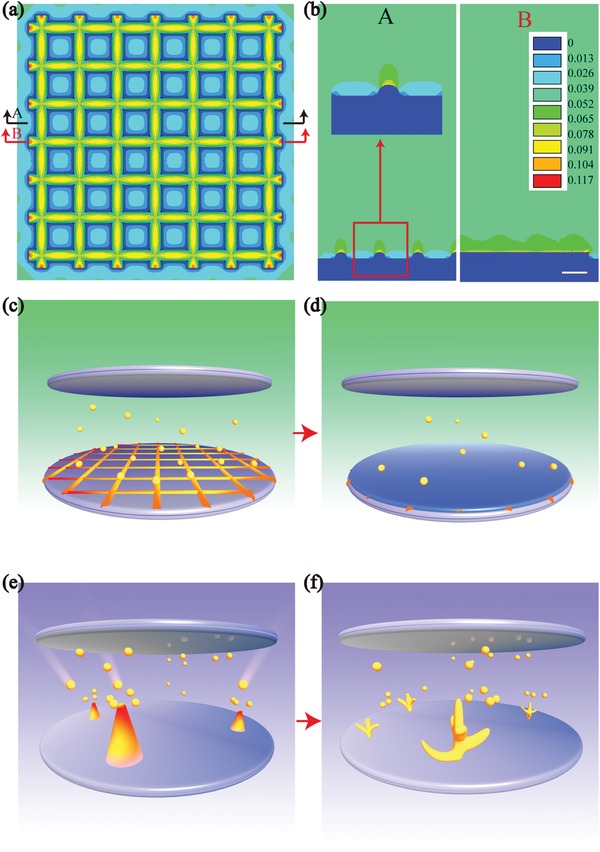
The simulation results of the electric field distribution of the LiB‐Li alloy. a) Top view, b) cross‐sectional view from different perspectives (A: left side and B: right side) of the simulation results. c,d) Schematic diagrams of Li deposition behavior on the LiB–Li composite surface. e,f) Schematic diagrams of Li dendrite evolution. The scale length in (b) is 1 µm.

The uneven electric field distribution is regarded as a main driving force for the uncontrolled Li dendrites growth.^27^ The currently proposed porous and conductive 3D LiB skeleton is expected to decentralize the electric field, reduce the local current density and regulate the Li^+^ deposition. To elucidate the effect of the LiB skeleton on electric field distribution, electrical conduction model via finite element analysis (FEA) simulation is conducted. (Note that the simulation is concentrated mainly on the electric field distribution on the composite surface during the initial Li deposition process.) The results are shown in **Figure** **5** (details and Figure S7 are shown in the Supporting Information). As illustrated in Figure 5a–d, the uniformly distributed electric field generated from the LiB skeleton facilitates the uniform deposition of Li, inhibiting the formation of Li dendrite. Moreover, the remaining Li(Mg) solid solution with high Mg concentration will also help to guide the uniform deposition of Li^+^ due to the strong affinity to Li as mentioned earlier. In comparison, the Li tips on the Li surface would eventually evolve into Li dendrites due to the inhomogeneous incoming Li^+^ flux (Figure 5e,f).

**Figure 6:**
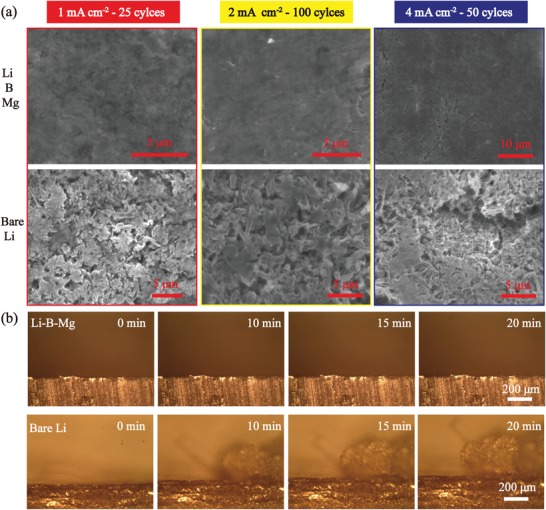
a) The SEM pictures of the Li–B–Mg composite and the pure Li foil after cycling under different current densities. b) The in situ observation of Li plating on the Li–B–Mg composite and pure Li foil under the current density of 3 mA cm^−2^ in carbonate‐based electrolyte.

The morphology evolution of Li–B–Mg composite and pure Li after electrochemical cycling are characterized by SEM to further investigate the superior dendrite inhibition of Li–B–Mg composite and the results are presented in **Figure** **6**a. The first image in Figure 6a shows a relatively smooth and flat surface of the composite after 25 cycles cycled at 1 mA cm^−2^ with a fixed capacity of 0.5 mA h cm^−2^. This indicates that the 3D Li–B–Mg composite can successfully accommodate the electrochemically deposited Li. By contrast, a great deal of dendrites of several micrometers in diameter can be observed on the surface of Li foil (second row in Figure 6a). These entangled dendrites grow randomly and form a porous layer on planar Li foil (Figure S8, Supporting Information). It has been reported that this porous layer would undoubtedly bring detrimental effect to Li^+^ diffusion and contribute significantly to the generation of electrochemically “dead Li.”^28^ The Li–B–Mg composite electrode also shows superior dendrite inhibition ability at increased cycle numbers and current density (the middle and right columns in Figure 6a).

In situ optical microscopy was employed to record the Li electrodeposition on the 3D Li–B–Mg composite and pure Li electrode in the carbonate‐based electrolyte. The results are shown in Figure 6b and Videos S1 and S2 in the Supporting Information. From Figure 6b, one can hardly observe dendrite formation on Li–B–Mg electrode surface, as shown in the first row of Figure 6b. However, tremendous amount of Li cluster appears and it grows rapidly into irregular bulges during electrochemical plating. These results illustrate that the Li–B–Mg electrode is indeed capable of maintaining relatively flat surface by inhibiting dendrite growth during cycling.

EIS measurements were also carried out to examine the interfacial stability of the Li–B–Mg anode and pure Li anode over the range of 0.01–10^5^ Hz during cycling. Figure S9 (Supporting Information) shows the results of the interfacial resistance measurements after different cycles at 0.5 mA cm^−2^ with a fixed capacity of 0.5 mA h cm^−2^. It can be clearly observed from Figure S9 (Supporting Information) that the Li–B–Mg composite assembled symmetrical battery exhibits a stable and ultralow interfacial resistance of less than 15 Ω during the cycling after the first cycle. On the contrary, pure Li assembled symmetrical battery displays erratic resistance behavior, indicating that an unstable interface between metallic Li and the electrolyte has been formed.

### 2.5 Full Batteries Paired with LiCoO_2_

**Figure 7:**
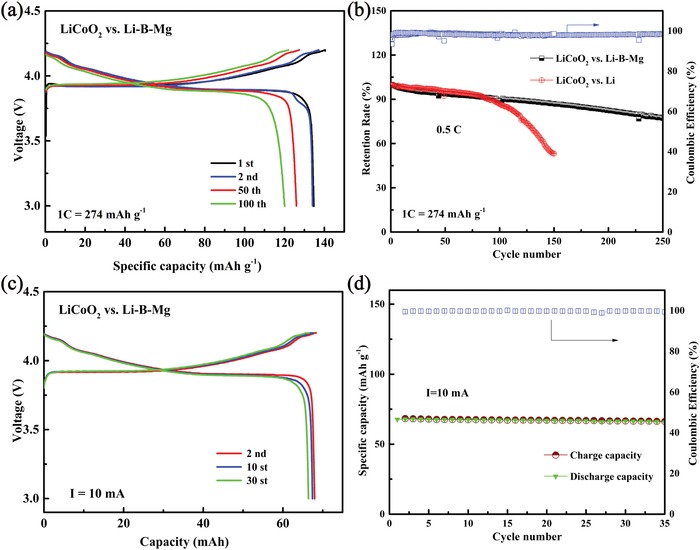
The charge–discharge curves and cycling performance of a,b) coin‐type batteries and c,d) pouch‐type batteries using LiCoO_2_ as cathode in carbonate‐based electrolyte.

To explore the practical application of the Li–B–Mg composite, coin‐type full battery using commercial LiCoO_2_ as cathode was assembled. The full battery employed carbonate‐based electrolyte and its voltage profiles within the voltage range of 3.0–4.2 V at different cycles are presented in **Figure** **7**a. Under the current density of 0.5 C (1 C = 274 mA h g^−1^), LiCoO_2_|Li–B–Mg full battery displays a discharge specific capacity of 134.7 mA h g^−1^ with a distinct voltage plateaus at ≈3.9 V in the first cycle. In the subsequent cycles, the voltage plateau remains unchanged. Figure 7b compares the cycling stability of the batteries assembled respectively with the Li–B–Mg composite and pure Li anodes at 0.5 C. The initial discharge specific capacity of the LiCoO_2_|Li full battery approximates to that of the LiCoO_2_|Li–B–Mg full battery (134.3 vs 134.7 mA h g^−1^). However, the LiCoO_2_|Li full battery decays quickly during the 100 cycles while the LiCoO_2_|Li–B–Mg full battery exhibits a much longer cycling life (250 cycles with a capacity retention of 77.3%). The pouch‐type battery was also assembled to test the practical performance of the Li–B–Mg composite. Its first discharge capacity is 67.657 mA h (≈6 mA h cm^−2^). In addition, the pouch‐type battery demonstrates a similar charge–discharge curve to that of the coin‐type battery (Figure 7c) and a very stable cycling over 35 cycles is shown in Figure 7d. Meanwhile, its Coulombic efficiency maintains over 99.7%, verifying its feasibility for practical application.

Safety concerns of LMBs are of paramount importance in practical applications. A series of safety tests of the pouch‐type battery using the Li–B–Mg composite as anode, namely, the acupuncture, impact and overcharge tests, were conducted after preformation procedure. Among them, the acupuncture test is the simulation of a battery under the circumstance of a forced internal short circuit. Video S3 (Supporting Information) recorded the process of the acupuncture test, from which no instant explosion and no open fire can be observed after the needle puncture (the inset on the upper right in Figure S10A in the Supporting Information). The pouch battery also withstood the impact test without explosion and open fire (Video S4 and Figure S10B, Supporting Information), which is aimed at simulating a heavy object impacting the power supply. Resistance to overcharge is also an essential aspect of the safety properties for monomer battery so an overcharge test has been conducted and the results are shown in Figure S10C (Supporting Information). Although it can be observed that swelling has occurred during the rise of voltage yet no explosion nor smoke occurred even when the voltage has reached to 10 V. This experiment demonstrates a higher safety characteristic of the LiCoO_2_|Li–B–Mg pouch battery.

## 3 Conclusion

Li–B–Mg composite with in situ formed 3D LiB fiber network is successfully manufactured and introduced as a potential anode for LMBs. On the one hand, the fibrillar conductive LiB skeleton not only contributes to the reduction of the local current density, but also acts as a host for the storage of Li; on the other hand, the stronger affinity to Li of the skeleton due to the introduction of Mg regulates the incoming Li^+^ flux and helps to reduce Li ion concentration gradient during Li deposition. Besides, Li(Mg) alloy with large solid solution is also beneficial to maintain the integrity and stability of the structure during Li electrodissolution/electrodeposition. As a result, the proposed 3D Li–B–Mg composite anode shows less volume change and a dendrite‐free feature during electrochemical cycling. In addition, the Li–B–Mg composite assembled symmetrical battery achieves a long and stable cycle lifespan of more than 500 h at 0.5 mA cm^−2^ with a low overpotential. Moreover, full batteries assembled with the proposed 3D Li–B–Mg composite display improved electrochemical performance compared to that assembled with pure Li. The pouch‐type battery also shows outstanding safety properties. Therefore, on the basis of current research, it is suggested that the Li–B–Mg composite is a promising candidate to substitute Li metal anode in LMBs and it may significantly propel the advancement of LMB technology from laboratory research to industrial commercialization.

## 4 Experimental Section

*Materials Preparation*: The Li metal ribbon (99.9%) was purchased from Wenyuan (Wuhan) Advanced Materials Co., Ltd. and it was rolled into a 0.2 mm thick foil. The Li–B–Mg composite was fabricated by the smelting reaction.^20^, ^29^ In brief, proportional lithium, boron, and magnesium were put in an iron crucible and then they were experienced two exothermic reactions in Ar (350 and 530 °C) under vigorous stirring condition. Afterward, the obtained composite was rolled into a 0.2 mm thick foil and 16 mm diameter disks were punched out therefrom as anode electrodes for LMBs. The control sample Li–B composite was also prepared without Mg added. Table S1 (Supporting Information) demonstrates the mass ratios of raw materials.

*Materials Characterization*: Phase structure was characterized via XRD analysis by Rigaku Deskto X‐ray diffractometer (Japan) using Cu Kα radiation (λ = 1.54056 Å). The XRD samples were wrapped in transparent tape and then fixed on the specimen holder. To avoid oxidation, the whole procedure was conducted in Ar‐filled glove‐box (O_2_ and H_2_O < 0.5 ppm). The XRD spectrum of the tape was also tested. The morphology was characterized by SEM (Hitachi SU8010, Japan). The SEM samples were loaded into a transfer vessel under Ar protection which could be opened inside the SEM. The ex situ XRD and SEM samples were obtained from the disassembled batteries and they were washed with dimethyl carbonate (DMC) followed by subsequent drying in Ar‐filled glove‐box overnight. To study the affinity of Li and the skeleton, the round composite ingot and Li plate were respectively heated on the Al foil on a hot plate to 300 °C in Ar‐filled glove.

*Electrochemical Measurement*: Electrochemical tests were carried out in CR2016 coin batteries. The electrolyte was 1 m LiPF_6_ in ethylene carbonate (EC), ethyl methyl carbonate (EMC), dimethyl carbonate (DMC) (1:1:1, volume) with the additive of vinylene carbonate (VC). All battery employed the Celgard 2400 separator. The measurement of the free Li capacity in composite was done by charging the battery to 1 V at 0.2 mA cm^−2^ with the pure Li as the counter and reference electrode. In the symmetrical battery type, either Li–B–Mg composite disks or pure Li on both sides were used. Galvanostatic experiments were conducted on a battery testing system (LANHE CT 2001A, Wuhan LAND electronics Co., P.R. China) under a current density of 0.5 mA cm^−2^. A symmetrical cuvette‐type optical battery was assembled to in situ observe the surface change of Li–B–Mg composite and pure Li under 3 mA cm^−2^ by a metallurgical microscope (Caikon Optical Instrument DMM‐330C, with 8.9 mm extra‐long working distance 10× objectives^30^). The electrochemical impedance spectroscopy (EIS, Princeton PARSTAT 4000, AMETEK Co. Ltd) was tested in the frequency range of 10^5^–0.01 Hz. Commercialized LiCoO_2_ powder used for the cathode in full batteries (both coin‐type and pouch batteries) was purchased from Hunan Shanshan Advanced Energy Co., Ltd. For coin‐type full battery, the cathode slurry comprising LiCoO_2_, super P and polyvinylidene fluoride (PVDF) at the weight ratio of 8:1:1 was coated onto the Al foil. The slurry coated Al foil was cut into a 12 mm diameter disk after drying overnight under vacuum at 120 °C. The active substance loading of cathode is 1.40 mg cm^−2^. In the pouch battery the designed capacity of the anode is 50% more than that of cathode and the assembly as well as the safety property tests was conducted in Dongguan Juda Electronics Co. LTD (Guangdong, China). Before electrochemical performance testing, the pouch batteries underwent a preformation procedure, which was performed under two constant‐current charging and a constant‐voltage charging processes with the upper limit voltages of 3.3 V, 3.6 and 4.2 V respectively.

*Computational Methods*: The calculations of the adsorption energy among Li, LiB, and Mg were performed using DFT implemented in the Vienna ab initio simulation package (VASP).^31^ The projector augmented wave (PAW) method^32^ and the generalized gradient approximation (GGA)^33^ in the form of Perdew, Burke, and Ernzerhof (PBE) were used in this work. The cut‐off energy was set to be 520 eV. The Brillouin zones were sampled with 2 × 2 × 1 Monkhorst–Pack meshes. The atomic position was fully relaxed until the maximum force on each atom was less than −0.02 eV Å^−1^ and 10^−6^ eV. A vacuum space of 20 Å was inserted along the *z* direction to avoid any interactions between the periodically repeated images.

The ANSYS FEA package was employed to simulate the effect of the skeleton on electric field distribution in LiB–Li system based on the electrical conduction model.^34^ The computational work were mainly focused on the surface of the composite and details are depicted in the Supporting Information.

## Conflict of Interest

The authors declare no conflict of interest.

## Supporting information

Supporting Information

Supplemental Video S1

Supplemental Video S2

Supplemental Video S3

Supplemental Video S4
