# Supplementary material for: Mg Doped Li–LiB Alloy with In Situ Formed Lithiophilic LiB Skeleton for Lithium Metal Batteries
Source: Adv Sci (Weinh). 2020 Jan 9;7(6):1902643. doi: 10.1002/advs.201902643 (PMC7080552; doi:10.1002/advs.201902643)
Supplement: Supplementary file 1 — Supporting Information [file ADVS-7-1902643-s001.pdf]

Copyright WILEY-VCH Verlag GmbH & Co. KGaA, 69469 Weinheim, Germany, 2020.

ADVANCED SCIENCE Open Access

Supporting Information

for *Adv. Sci.,* DOI: 10.1002/advs.201902643

Mg Doped Li–LiB Alloy with In Situ Formed Lithiophilic LiB Skeleton for Lithium Metal Batteries

Chen Wu, Haifeng Huang, Weiyi Lu, Zengxi Wei, Xuyan Ni, Fu Sun, Piao Qing, Zhijian Liu, Jianmin Ma, Weifeng Wei, Libao Chen, Chenglin Yan,* and Liqiang Mai**

Supporting Information

Mg doped Li-LiB alloy with in-situ formed Lithiophilic LiB Skeleton for Lithium Metal Batteries

Chen Wu, Haifeng Huang, Weiyi Lu, Zengxi Wei, Xuyan Ni, Fu Sun, Piao Qing, Zhijian Liu, Jianmin Ma, Weifeng Wei, Libao Chen, Chenglin Yan*, Liqiang Mai**

Simulation models and setup

The finite element analysis (FEA) simulation was used to calculate the electric field distribution on the surface of LiB-Li alloy in this work. To reduce the calculation workload, the model is simplified by assuming 1) the LiB fibers are in orthogonal design and 2) Mg element is not taken into account. Both the length and the height of the entire model were 11 µm. The Li layer in the bottom is 1µm thick and the electrolyte was set to be 10 µm. The LiB fibers were set to be halfly embedded into the Li layer with a diameter of 500 nm. The distance between the neighboring two LiB fibers was about 1 µm. The electrical resistivity used for the Li metal can be found in the published literatures.[1] For pure LiB fibers, there is no related reports and it is difficult to measure its electrical resistivity due to its high reaction activity and brittleness after Li is stripped. Therefore, a composition ratio of $Li_{0.5}B_{0.5}$ was assumed to calculate its electrical resistivity. In practice, the LiB fibers are wrapped in a layer of Li during the stripping process

and the LiB fibers are always in the Li-rich state, so the different electrical resistivity of 12.5×10^(-8) Ω·m and 23×10^(-8) Ω·m are all considered.[1] In addition, an extreme value of 1.7 ×10^6 Ω·m (the electrical resistivity of pure B) is also considered. The ionic conductivity of the 1 M $LiPF_6$ in EC, EMC and DMC (1:1:1, volume) with the additive of VC was set to be 10 mS/cm^2.

Table S1. The ratios of the original materials

Materials	Mass percent (Li-B-Mg)	Mass percent (Li-B)
Li	70	74
B	26	26
Mg	4	-

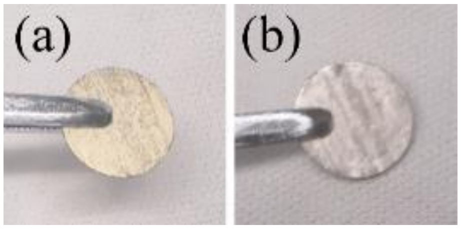

Figure S1. The digital images of (a) the Li-B-Mg composite and (b) pure Li plate.

The mass percent of the LiB in composite:

Assuming the total mass of the raw material is 100 g.

The molar masses of the Li, B and LiB are respectively 6.941, 10.811 and 17.752 g mol^{-1}.

The formed amount of the LiB phase is 26 / 10.811 * 17.752 = 42.693 g.

The remaining amount of the Li is 70 – (26 / 10.811 * 6.941) = 53.307 g.

The molar number of the remaining Li is 53.307 / 6.941 = 7.680 mol

The molar number of the Mg is 4 / 24.31 = 0.1645 mol

So the composition of the solid solution is approximately $Li_{0.98}Mg_{0.02}$

Suppose only the remaining Li can participate in the dissolution and deposition, so the specific capacity of the composite is 3860 mA h g^{-1} * 53.307 g / 100 g = 2057.650 mA h g^{-1}. However, not all Li can contribute to the capacity due to the presence of Mg. Therefore, it is necessary to make actual test of the available Li capacity.

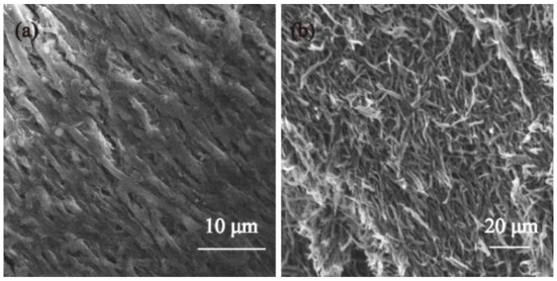

Figure S2. The SEM images of the composite after soaking in the THF solution containing naphthalene (10 wt. %) for 3 h. (a) surface (b) the cross section.

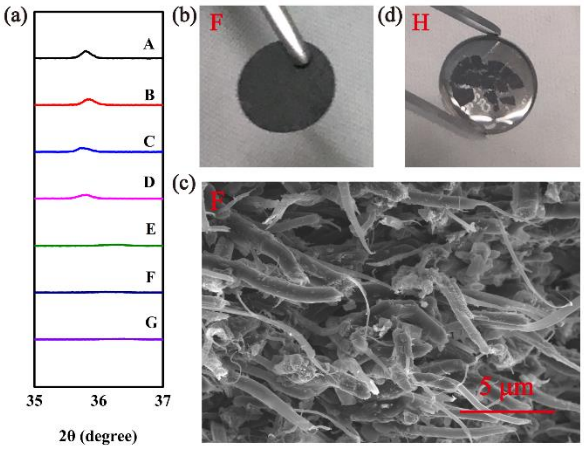

Figure S3. (a) The enlarged details of the peak centered around 36° during the Li dissolution from the composite. (b) The digital image and (c) the cross-sectional SEM of the Li-B-Mg composite plate after Li stripping at F point. (d) The digital image of the Li-B-Mg composite plate after Li stripping over 1 V (H point).

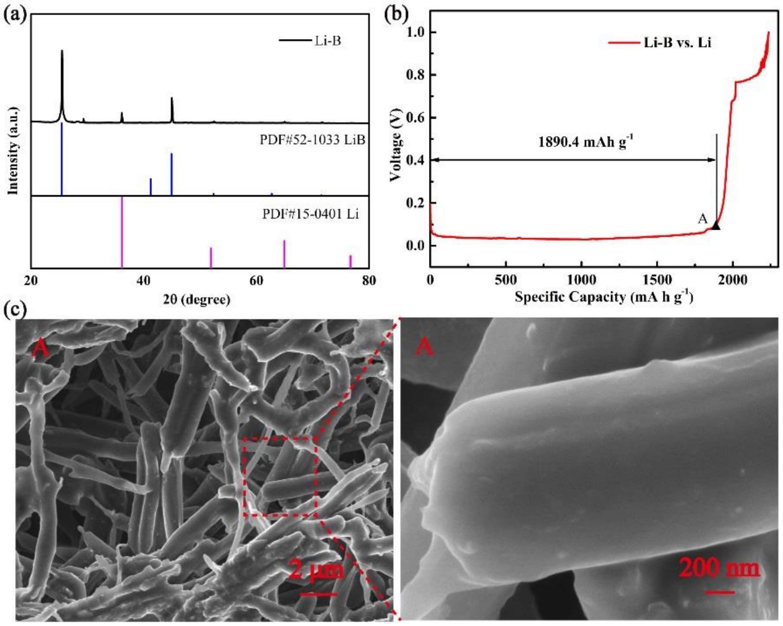

Figure S4. (a) The XRD of Li-B (b) Voltage profile of the electrochemical Li stripping from the Li-B anode to 1 V versus Li^+/Li. (c) The morphology of Li-B anode after Li stripped to A stage marked in (b).

The theoretical thickness change of the Li metal after Li stripping.

The theoretical capacity of the 0.6 mm-thick Li:

$V = 0.06\ cm * 3.14 * (1.60 / 2\ cm)^2 = 0.12\ cm^3$

$Capacity = V * \rho_{Li} * 3860\ mA\ h\ g^{-1} = 247.35\ mA\ h\ g^{-1}$

The reduced thickness after stripping 15.30 mA h Li:

$15.30 / 247.35 * 600\ \mu m = 37.11\ \mu m$

The thickness reduction when stripping 60.00 mA h Li:

$60.00 / 247.35 * 600\ \mu m = 145.54\ \mu m$

So, the theoretical thickness reduction is 37.11 μm when stripping 15.30 mA h (25 %) Li and 145.54 μm for 60.00 mA h Li.

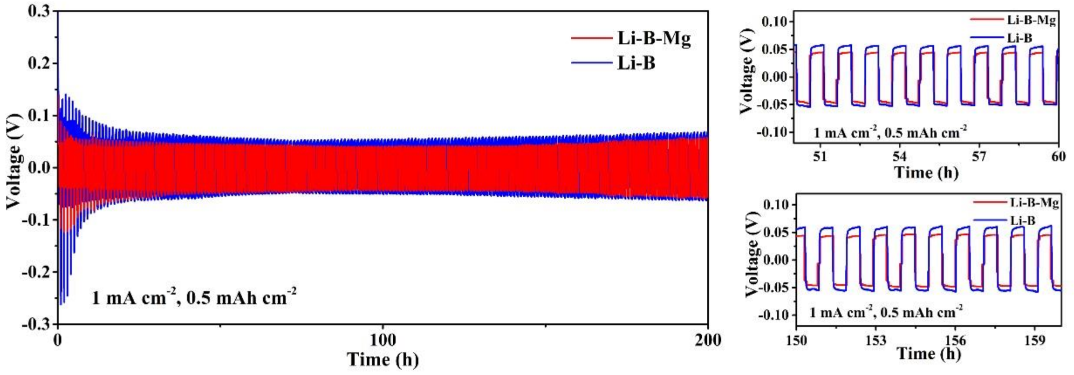

Figure S5. The voltage-time profiles and detailed overpotential curves of the Li-B-Mg and Li-B composite assembled symmetrical batteries at 1 mA cm^{-2} with a fixed Li stripping/plating capacity of 0.5 mA h cm^{-2}.

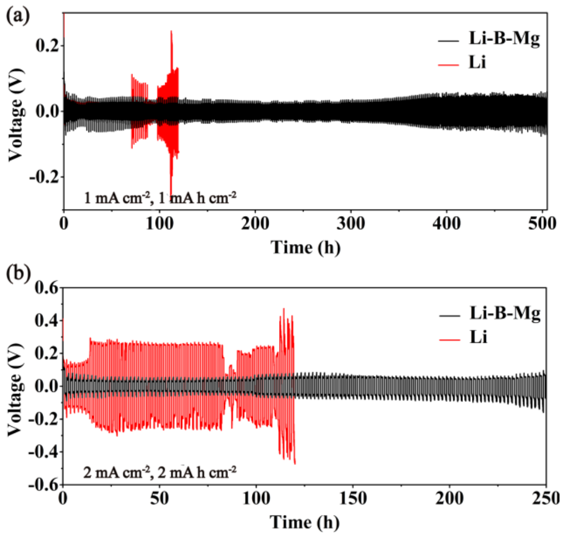

Figure S6. The electrochemical performance comparison of the Li-B-Mg composite-based and pure Li-based symmetrical batteries in ether-based electrolyte (1 M LiTFSI in DOL/DME with 2 % $LiNO_3$)

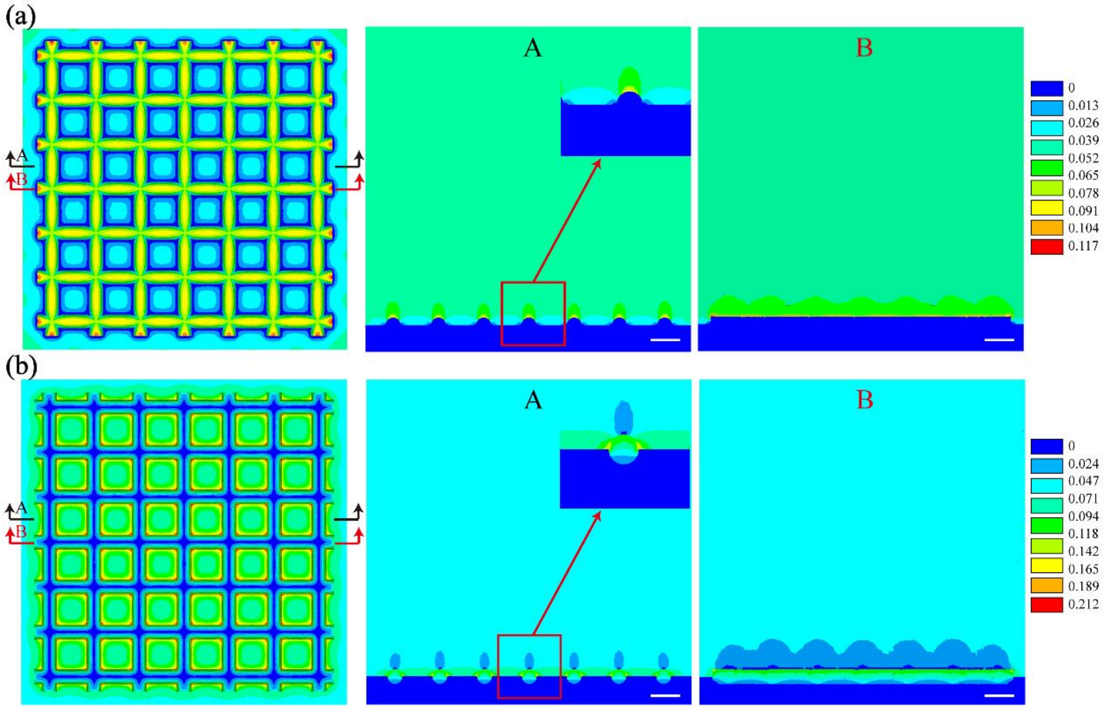

Figure S7. The simulation results of the electric field distribution in the LiB-Li alloy with different electrical resistivity of the skeleton. (a) 23 × 10^ (-8) Ω·m. (b) 1.7 × 10^6 Ω·m. The scale length is 1 μm.

When the electrical resistivity of the skeleton is set to be 23×10^(-8) Ω·m (Figure S5a), the electrical field distribution is similar to that shown in Figure 5a-c. But the situation is different when the electrical resistivity increases to 1.7 × 10^6 Ω·m. This implies that the electrical resistivity has a significant impact on the electric field distribution.

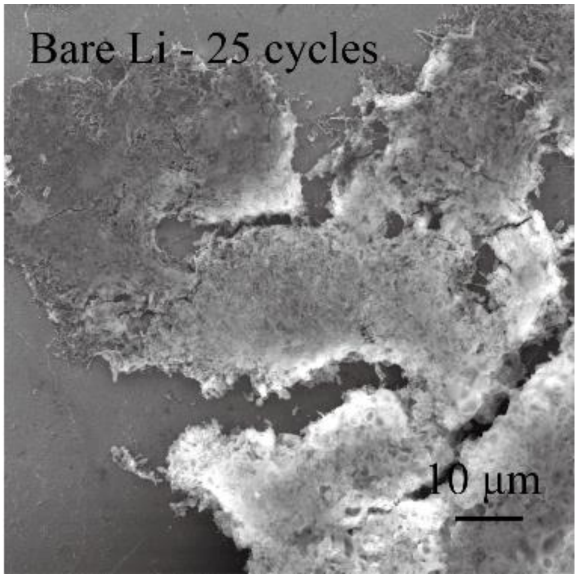

Figure S8. The SEM picture of the surface of pure Li after 25 cycles .

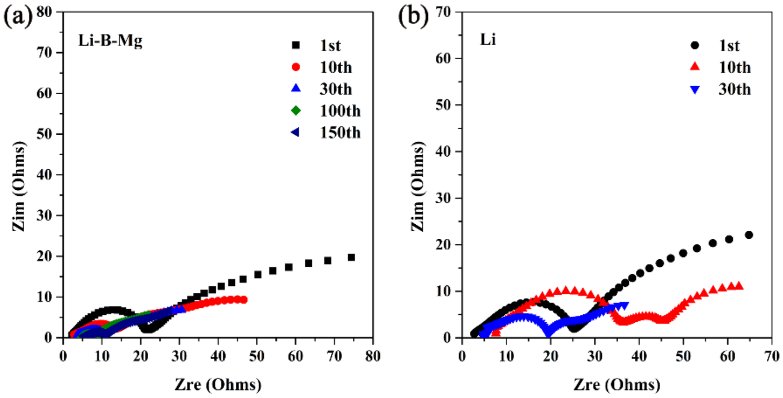

Figure S9. EIS of (a) the Li-B-Mg composite-assembled and (b) Pure Li foil assembled symmetrical battery after different cycles.

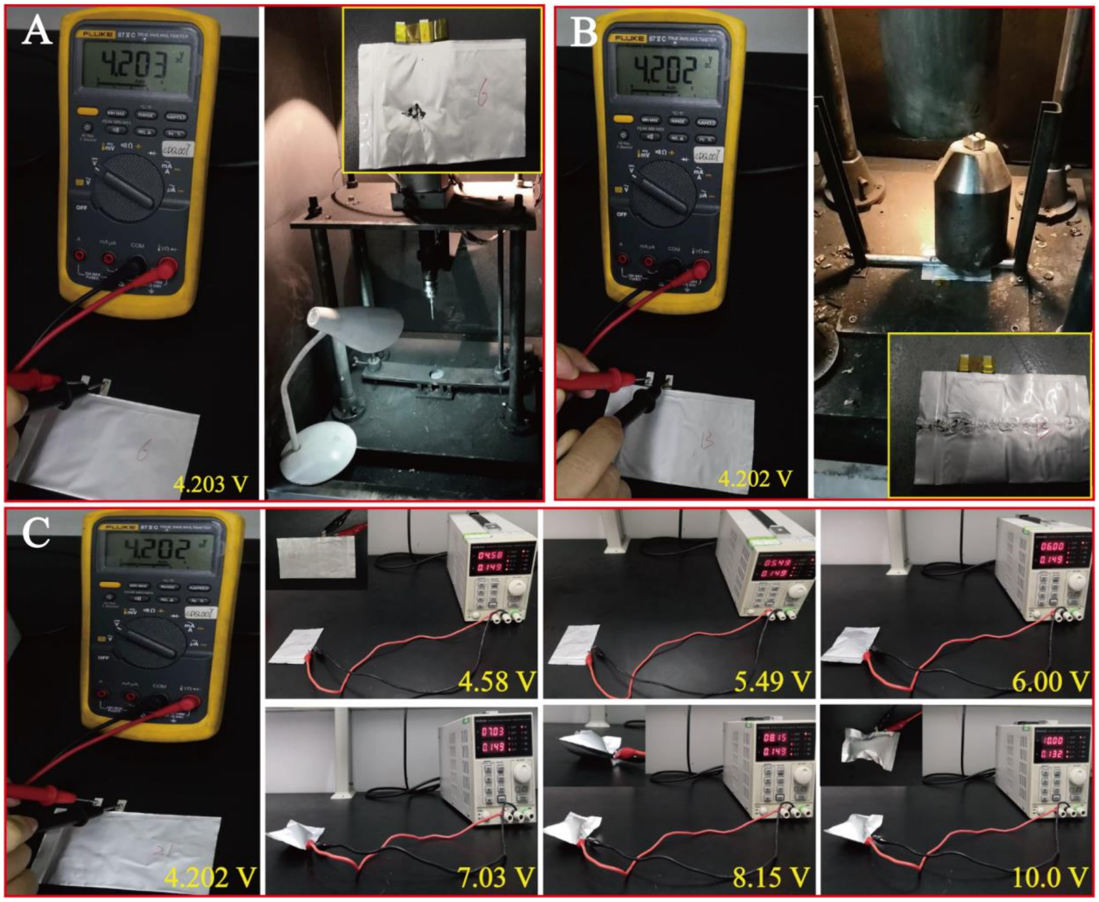

Figure S10. Safety performance tests of the pouch-type battery. (A) Acupuncture test. (B) Impact test. (C) The overcharge test.

Li-B-Mg-10s.mp4

Video S1. The *in situ* observation of the Li deposition process on Li-B-Mg electrode

Li-10s.mp4

Video S2. The *in situ* observation of the Li deposition process on pure Li electrode

Acupuncture Test.mp4

Video S3. The acupuncture test.

Impact Test2.mp4

Video S4. The impact test.

Reference

1. M. A. Mitchell, R. A. Sutula, *J. Less-Common Met.* **1978**, *57*, 161.

2. M. Smart, B.Ratnakumar, S. Surampudi, **1998**.
